# Diabetes and Risk of Parkinson's Disease: An Updated Meta-Analysis of Case-Control Studies

**DOI:** 10.1371/journal.pone.0085781

**Published:** 2014-01-21

**Authors:** Lin Lu, Deng-lei Fu, Hui-qin Li, Ai-ju Liu, Ji-huang Li, Guo-qing Zheng

**Affiliations:** Department of Neurology, the Second Affiliated Hospital of Wenzhou Medical University, Wenzhou, China; CSIR-Central Drug Research Institute, India

## Abstract

**Background:**

Whether diabetes increases the risk of Parkinson's disease (PD) is still inconclusive. The objective of this updated meta-analysis is to synthesize evidence from case-control studies that evaluated the association between diabetes and the risk of PD.

**Methods:**

Seven databases were searched to identify case-control studies that evaluated the association between diabetes and PD. The methodological quality of included studies was assessed using Newcastle-Ottawa scale. All data were analyzed using Review Manager 5.1 software. Subgroup analyses were also adopted, according to stratification on gender, geographic location, source of the control group, smoking, anti-diabetes drug prescription and duration of DM.

**Results:**

Fourteen studies fulfilled inclusion criteria for meta-analysis, yielding a total of 21395 PD patients and 84579 control subjects. Individuals with diabetes were found to have a negative association with future PD (OR 0.75; 95% CI 0.58–0.98) in spite of significant heterogeneity. In subgroup analyses, the negative correlation was still found in studies from North America, non-PD control groups from general population, never smoking individuals, and DM ascertainment based on questionnaire or self-report. Stratification of gender and DM duration showed no significant association. No association was also found in European and Asian individuals, hospital-based controls, ever smoking subjects, DM assessment by medical record or physician diagnosis, and insulin prescription for DM.

**Conclusion:**

Evidence from case-control studies suggested that diabetic individuals may have a decreased incidence of PD despite significant heterogeneity. More researches are warranted to clarify an understanding of the association between diabetes and risk of PD.

## Introduction

Parkinson's disease (PD) is the second most common chronic neurodegenerative disorder after Alzheimer disease, and affects more than 1% of the elderly population worldwide [1]. Recent literature suggested that diabetes mellitus (DM) has been associated with PD, and they have shared similar pathogenic pathways [2], [3]. Genetic and environmental factors cause dysregulation in common pathways that lead to neurodegeneration and diabetes [2]. Moreover, insulin and dopamine may exert reciprocal regulation between PD and diabetes [3]. However, the relationship between diabetes and PD was inconsistent with several epidemiological studies, ranging from a positive association to a null, or even inverse association [4]–[12].

A recent systematic review and meta-analysis on the risk of PD associated with diabetes has been published by Cereda et al. [13], and its conclusion suggested that diabetes was a risk factor for PD according to data from 4 cohort studies. However, no association was found between diabetes and PD based on data from 5 case-control studies. An update of risk estimates from 5 cohort studies was also conducted by Cereda et al. [14], suggesting that diabetes may be considered a risk factor for future PD. However, there was little evidence on this association because of the significant heterogeneity between studies [15]. Thus we conducted an updated systematic review, which incorporated more recent case-control studies, to further determine whether prior onset of diabetes contributes to the risk of PD.

## Research Design and Methods

We performed a systematic review of the published literature based on the guidelines for reporting of the Meta-analysis of Observational Studies in Epidemiology (MOOSE) [16]. Results are reported according to the recently published PRISMA guidelines [17].

### Eligibility criteria

We included those studies that met all of the following criteria: (1) reported separately relevant risk statistics for PD by antecedent diagnosis or characterization of diabetes based on case-control design; (2) one of the exposure of interest was DM; (3) the outcome of interest was PD; and (4) reported odds ratios (ORs) or risk ratios (RRs), with their 95% confidence intervals (CIs) for the exposure, or provided sufficient information to calculate them.

Studies were excluded if they were any of the following: (1) case reports, review articles, editorials, and clinical guidelines; (2) studies that did not provide effect estimates in OR or RR, or did not allow the computation of such effect estimates, as well as only provided an effect estimate with no means to calculate a CI; (3) studies used parkinsonism diagnoses as the outcome; (4) associations considered with non-preceding PD.

To evaluate studies' eligibility for inclusion, titles, abstracts, and articles were reviewed independently by LL and FDL. Discrepancies were resolved by consensus or by a third reviewer (ZGQ). Articles or reports from non-peer-reviewed sources were not included in this meta-analysis. In the event of multiple publications from the same study participants, we included only the one with the most recently detailed information for both outcome and exposure in the systematic review.

### Search methods

A computerized literature search was conducted using PubMed, Web of Science, Scopus, Google Scholar, Chinese National Knowledge Infrastructure (CNKI),VIP Journals Database and Wanfang database until May 2013 for studies of the association between diabetes and PD (see also Appendix S1). We also hand-searched bibliographies of retrieved papers for additional references, and contacted experts in the field for any unpublished studies. The researches and studies included were not limited by publication date, country, or language.

### Data collection and analysis

#### Selection of studies

Two review authors (LL, FDL) identified possible studies, and assessed the methodological quality of included studies independently, with disagreement settled by discussion with ZGQ.

#### Data extraction

Two investigators (LL, FDL) independently performed the data extraction on study characteristics, including the first author's last name, publication year, source of study, participant characteristics (age, sex, geographical location etc.), diagnoses of cases, method of ascertainment of diabetes, sample size, variables adjusted in the analysis, and the risk estimates with corresponding 95% CIs, into a standardized data extraction form. We extracted the OR or RR estimate that was adjusted for the greatest number of potential confounders from each study. When differences were found, the third (ZGQ) would make the definitive decision for data extraction. Reasons for the exclusion of studies were noted.

### Quality assessment

The quality of the included studies was assessed by Newcastle-Ottawa Scale (NOS) [18]. The NOS, a star system allowing a semi-quantitative assessment of nonrandomized study quality, contained eight items that were categorized into three major components, including selection, comparability, and exposure (case-control studies) or outcome (cohort studies). The scale ranged from zero to nine stars, which the later represented the highest methodological quality.

### Data Synthesis and Analysis

We analyzed the data using Review Manager (version 5.1) for the association between diabetes and PD. Meta-analyses of the risk of PD outcomes were carried out generating pooled odds ratios (ORs) with 95% confidence intervals (CIs). Heterogeneity among studies was estimated using Cochran's Q test (reported with a x^2^-value and P-value) and I^2^ statistic [19], [20]. For the Q test, a P-value of less than 0.1 was used as an indication for the presence of heterogeneity. For the I^2^ statistic, as a measure of the proportion of total variation in estimates, is due to heterogeneity rather than chance. I^2^ values of greater than 50% were considered to denote substantial heterogeneity.

To explore the potential heterogeneity between studies, we conducted analyses stratified by gender, geographic region, source of the control, diabetes duration and prescription treatment, and we also evaluated the impact of adjustment for smoking on the association between diabetes and the risk of PD. Sensitivity analysis was performed by excluding each study individually to assess its influence on the overall result of the meta-analysis.

Publication bias was detected graphically using a funnel plot of a trial's effect size against the standard error. A two-tailed P value of less than 0.05 was considered to be statistically significant.

## Results

### Study selection

**Figure 1:**
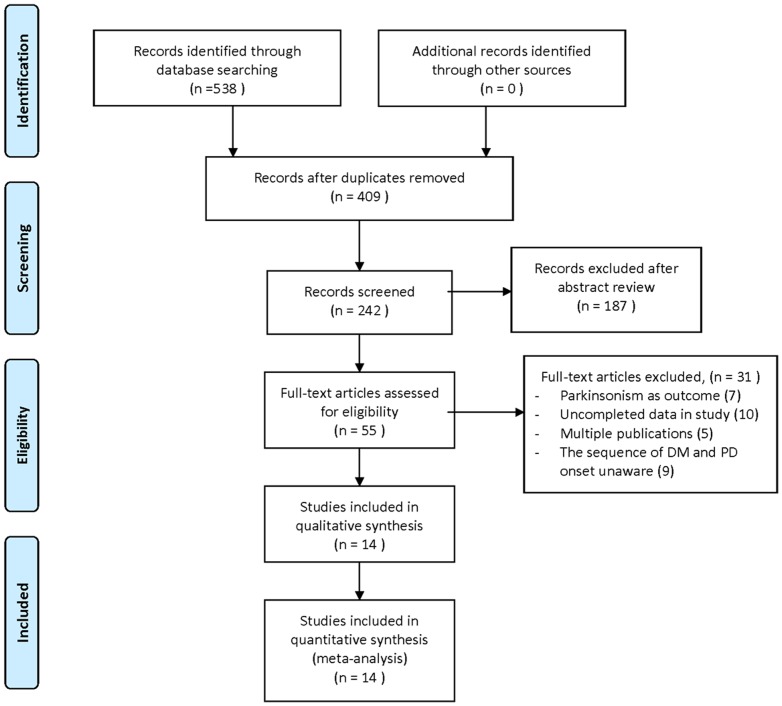
Flow chart of study selection.

We identified 242 literatures, of which 55 were considered to be of potential value and the full text was retrieved for detailed evaluation. Thirty-one of these 55 articles were subsequently excluded from the systematic review, of which 7 studies used Parkinsonism as outcome, 10 papers possessed uncompleted data that could not fulfill analysis, 5 exerted multiple publications, and the rest 9 studies provided no information on whether the onset of diabetes preceded the onset of PD. Thus, a total of 14 articles, which met the inclusion and exclusion criteria, were used in this systematic review. The screening process was summarized in a flow diagram (Figure 1).

### Study characteristics

This systematic review identified 105974 subjects, and most of the studies pointed out the specific male and female number in both PD patients and control subjects, with the exception of 3 studies for no information on gender [9], [21], [22]. The total number of PD cases in the included studies was 21395. Of these cases, 615 (2.9%) were diabetes. These cases were compared with 84579 non-PD individuals in the general population and hospital setting, of whom 1336 (1.6%) were diabetes. Four studies were conducted in North America [22]–[25], 7 in Europe [8], [10]–[12], [21], [26], [27], and the rest 3 in Asia [9], [28], [29]. Study size ranged from 140 to 82140 participants. PD was identified with register-based sources in all studies. Six studies ascertained diabetes with physician diagnosis or medical record [10], [12], [22], [23], [26], [27], 7 with by self-report or constructed questionnaire [8], [9], [11], [24], [25], [28], [29], and the study of Skeie et al. [21] with no mention. The majority of studies showed that the median ages at the onset of PD were over 60 years, with no description in 2 studies [9], [25]. None of the included studies differentiated types of diabetes distinctly, although Scigliano et al. noted that about 97% DM in cases and controls were type 2 diabetes [27]. Potential confounders were controlled in most of the studies, except in Kessler's study, where the confounders adjusted for were not indicated clearly. The detail characteristic of the included studies was listed (Table 1). The quality of included studies was moderate or good, varying from five to eight points (Table 2).

**Table 1:** Characteristics of the included studies.

Study	Study period	Resource	Location	Number of case (PD) subjects (M/F)	Mean age at onset PD (mean duration)	Number of prior DM in cases	Number of control subjects (M/F)	Source of the control	Number of DM in controls	Definition of PD	DM assessment	OR (95% CI) for cases	Adjustment variables
Skeie et al., 2013 [21]	2004–2006	Norwegian ParkWest study	Norway	212 (-)	67.3±9.8 (-)	18	175 (-)	General population	8	Gel criteria	Not stated	1.94 (0.82–4.57)	Age, sex
Savica et al., 2012 [23]	1976–1995	Rochester Epidemiology Project	U.S.	196 (121/75)	71 (2–73 years)	13	196 (121/75)	General population	17	Diagnostic criteria	Physician-diagnosed DM or use of glucose- lowering medications	0.77 (0.37–1.57)	Age, sex
Schernhammer et al., 2011 [26]	2001–2006	Danish Hospital Register	Denmark	1931 (1121/810)	72.2±10.2 (-)	126	9651 (5603/4048)	General population	482	ICD-10 code G20, ATC code N04B	ICD codes and ATC codes	1.36 (1.08–1.71)	Age, sex, and COPD
Miyake et al., 2010 [29]	2006–2008	11 hospitals in Japan	Japan	249 (93/156)	68.5±8.6 (within 6 years)	10	368 141/227)	Hospital setting	39	UK PD Society Brain Bank clinical diagnostic criteria	self-administered questionnaires	0.38 (0.17–0.79)	Age, sex, smoking, area of residence, BMI, education, leisure-time exercise, dietary in take of energy, cholesterol, vitamin E, alcohol and coffee, and the dietary glycemic index
Rugbjerg et al., 2009 [12]	1986–2006	Danish Hospital Register	Denmark	13695 (7423/6272)	73.0 (-)	48	68445 (37101/31344)	General population	223	ICD-8 code 342 and ICD-10 code G20	Medical record	1.1 (0.8–1.5)	Age, sex
D'Amelio et al., 2009 [11]	-	Neurological Department, Palermo	Italy	318 (153/165)	60.8 (5.9 years)	13	318 (153/165)	General population	31	2 out of 4 cardinal signs, progressive course, good response to anti-parkinsonian drugs	Semistructured questionnaire	0.4 (0.2–0.8)	Age, sex, education, BMI, occupational status, alcohol and coffee consumption, and smoking habit
Becker et al., 2008 [10]	1994–2005	General Practice Research Database, UK	U.K.	3637 (2167/1470)	90% aged >60 years (-)	291	3637 (2167/1470)	Hospital setting	308	OXMIS codes	Antidiabetes- drug use or diet recommendation in the medical record	0.95 (0.80–1.14)	Age, sex, BMI, smoking, diuretics, β-blockers, systemic steroids, and comorbidities
Scigliano et al., 2006 [27]	1970–1987	C. Besta Neurological Institute, Milan	Italy	178 (92/86)	58.1±11.4 (16 months)	6	534 (276/258)	Hospital setting	58	2 out of 4 cardinal signs, good response to L-DOPA	Discharge diagnosis	0.30 (0.13–0.72)	Age and sex
Powers et al., 2006 [24]	1992–2005	Group Health Cooperative database, Washington	U.S.	352 (217/135)	69 (-)	26	484 (298/186)	Hospital setting	61	Neurologist diagnosed, 2 out of 4 cardinal signs	Structured questionnaire	0.62 (0.38–1.01)	Age, ethnicity, education, and smoking habit
Leibson et al., 2006 [22]	1976–1995	Olmsted County, Minnesota	U.S.	197 (-)	70±11 (-)	18	197 (-)	General population	24	REP diagnostic index, diagnostic criteria	ICD-9-CM	0.7 (0.4–1.4)	Age, sex
Herishanu et al., 2001 [9]	1989–1995	PD clinic of Soroka University Medical Centre	southern Israel	93 (-)	-	11	93 (-)	Hospital setting	26	2 out of 4 cardinal signs, progressive course, good response to L-DOPA	Structured questionnaire (T2DM)	0.35 (0.15–0.75)	Age, sex
Morano et al., 1994 [8]	1989–1990	General Hospitals in Caceres, Spain	Spain	74 (33/41)	65.4±1.07 (-)	12	148 (66/82)	Hospital setting	18	Diagnostic criteria	Structured questionnaire	1.39 (p = 0.85)	Age, sex
Ho et al., 1989 [28]	-	10 Old Age Homes in Shatin and Tai Po	Hong Kong	35 (11/24)	65–87 (-)	6	105 (33/72)	Hospital setting	12	Diagnostic criteria	Structured questionnaire	1.6 (0.5–5.1)	Age, sex
Kessler, 1972	1967–1969	Private physician referrals in Baltimore area	U.S.	228 (122/106)	-	17	228 (122/106)	General population	29	Physician-diagnosed PD	Structured interview	0.84 (M); 0.30 (F)	None

ATC: Anatomical Therapeutic Chemical; COPD: Chronic Obstructive Pulmonary Disease;DM: Diabetes Mellitus; ICD-9: International Classification of Disease, 9th revision; M/F: Male/Female; OXMIS: Oxford Medical Information System; PD: Parkinson's Diseases; REP: Rochester Epidemiology Project.

**Table 2:** Newcastle-Ottawa Scale (NOS) assessment of the quality of the studies.

	Selection				Comparability	Exposure			
Study	Case definition adequate	Representativeness of the cases	Selection of controls	Definition of controls	Comparability based on design or analysis	Ascertainment of exposure	Same method of ascertainment for cases and controls	Non-response rate	Total scores
Skeie et al., 2013 [21]	★	★	★	★	★	☆	☆	☆	5
Savica et al., 2012 [23]	★	★	★	★	★★	★	★	☆	8
Schernhammer et al., 2011 [26]	★	★	★	★	★★	★	★	☆	8
Miyake et al., 2010 [29]	★	★	☆	★	★	☆	★	☆	5
Rugbjerg et al., 2009 [12]	★	★	★	★	★★	☆	★	☆	7
D'Amelio et al., 2009 [11]	★	★	★	★	★★	☆	★	☆	7
Becker et al., 2008 [10]	★	★	☆	★	★★	★	★	☆	7
Scigliano et al., 2006 [27]	★	★	☆	★	★★	☆	★	☆	6
Powers et al., 2006 [24]	★	★	☆	★	★	☆	★	☆	5
Leibson et al., 2006 [22]	★	★	☆	★	★★	★	★	☆	7
Herishanu et al., 2001 [9]	★	★	☆	★	★★	☆	★	☆	6
Morano et al., 1994 [8]	★	★	☆	★	★★	☆	★	☆	6
Ho et al., 1989 [28]	★	★	☆	★	★★	☆	★	☆	6
Kessler, 1972	★	☆	★	★	★	☆	★	☆	5

### Results of meta-analyses

**Figure 2:**
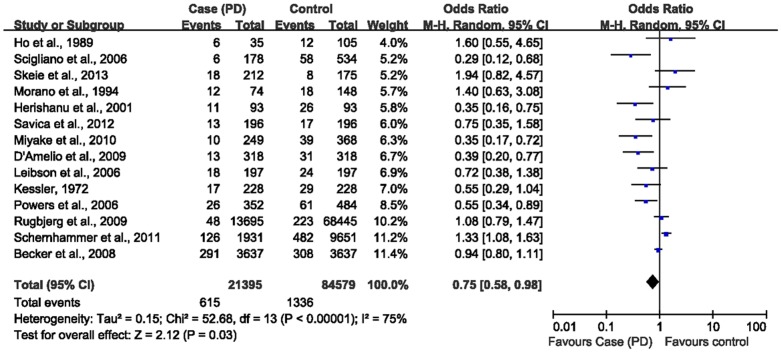
Forest plot of diabetes and risk of Parkinson's disease.

We conducted a primary meta-analysis with all the 14 identified studies that reported results on diabetes and PD incidence. The pooled summary OR was 0.75 (95% CI 0.58–0.98) in a random-effect model for PD patients, compared with non-PD individuals (Figure 2). There was significant heterogeneity among these studies (Q = 52.68, P<0.00001, I^2^ = 75%). To further elicit the association between diabetes and the risk of PD, subgroup analyses were adopted, according to stratification on gender, geographic location, source of the control group, smoking, anti-diabetes drug prescription and duration of DM (Table 3). There was no significant difference between cases and controls for the prevalence of DM in men and women separately [11], [23]–[26]. No statistical significance was found in subgroup analyses by DM duration [12], [23] and insulin prescription of DM [23], (Table 3). Oral anti-diabetes drug appeared to increase PD risk in Schernhammer's study (OR 1.37; 95% CI 1.10–1.71) [26]. Half of the included studies using general population-based controls reported inverse pooled estimate (OR 0.63; 95% CI 0.40–0.99) [8]–[10], [24], [27]–[29], while the other hospital-based controls reported no significant association (OR 0.88; 95% CI 0.62–1.25) [11], [12], [21]–[23], [25], [26]. A slightly intensified negative correlation was found between diabetes and the developing of PD in North America (OR 0.61; 95% CI 0.45–0.83) [22]–[25], whereas no significant association in Europe (OR 0.94; 95% CI 0.69–1.28) [8], [10]–[12], [21], [26], [27] and Asia (OR 0.54; 95% CI 0.23–1.27) [9], [28], [29]. Two studies showed that never smoking firmly strengthened the inverse association (OR 0.37; 95% CI 0.21–0.66), versus no significant difference in ever smoking individuals (OR 0.67; 95% CI 0.40–1.13) [11], [24] (Table 4).

**Table 3:** Univariate analysis: association between Parkinson's disease (PD) and diabetes preceding PD onset.

Study		Cases		Controls		OR (95% CI)	P
		Diabetes/tot.	%	Diabetes/tot.	%		
Savica et al., 2012 [23]	All individuals	13/196	6.6	17/196	8.7	0.67 (0.31–1.48)	0.32
	Gender						
	Male	7/121	5.8	12/121	9.9	0.57 (0.21–1.56)	0.28
	Female	6/75	8.0	5/75	6.7	1.05 (0.28–3.86)	0.95
	DM duration						
	0–9 years before index	5/196	2.6	9/196	4.6	0.51 (0.17–1.57)	0.24
	≥10 years before index	8/196	4.1	8/196	4.1	0.88 (0.30–2.54)	0.81
	Antidiabetes drug prescription						
	Insulin prescription	5/196	2.6	4/196	2.0	1.46 (0.38–5.58)	0.58
Schernhammer et al., 2011 [26]	All individuals	126/1931	6.5	482/9651	5.0	1.35 (1.10–1.65)	-
	Gender						
	Male	79/1121	7.0	305/5603	5.4	1.33 (1.03–1.72)	-
	Female	47/810	5.8	177/4048	4.4	1.38 (0.99–1.92)	-
	Age at PD onset						
	<60	17/257	6.6	29/1286	2.3	3.07 (1.65–5.70)	-
	≥60	109/1674	6.5	453/8365	5.4	1.24 (0.99–1.53)	-
	Antidiabetes drug prescription						
	Insulin prescription	19/1931	1.0	81/9651	0.8	1.22 (0.74–2.02)	-
	Oral antidiabetes drug	107/1931	5.5	401/9651	4.1	1.37 (1.10–1.71)	-
Rugbjerg et al., 2009 [12]	All individuals	48/13695	0.4	223/68445	0.3	1.1 (0.8–1.5)	-
	DM duration						
	5–9 years	31/13695	0.2	161/68445	0.2	1.0 (0.7–1.4)	-
	10-14 years	15/13695	0.1	51/68445	0.1	1.5 (0.8–2.6)	-
	≥15 years	2/13695	0.0	11/68445	0.0	0.9 (0.2–4.2)	-
D'Amelio et al., 2009 [11]	All individuals	13/318	4.1	31/318	9.8	0.4 (0.2–0.8)	0.007
	Gender						
	Male	8/153	5.2	17/153	11.1	0.4 (0.2–1.0)	0.05
	Female	5/165	3.0	14/165	8.5	0.4 (0.1–1.0)	0.05
	Age at interview						
	<66.7	8/180	4.5	17/178	9.6	0.4 (0.1–1.0)	0.05
	≥66.7	5/138	3.6	14/140	10.0	0.4 (0.2–1.0)	0.05
	Age at PD onset						
	<60.8	2/140	1.4	8/143	5.6	0.2 (0.1–1.1)	0.07
	≥60.8	11/178	6.2	23/175	13.1	0.5 (0.2–1.0)	0.04
	BMI						
	<26.1	4/179	2.2	9/173	5.2	0.4 (0.1–1.3)	0.1
	≥26.1	9/139	6.5	22/145	15.2	0.4 (0.2–0.9)	0.03
	Smoking						
	Ever	6/126	4.8	14/138	10.1	0.3 (0.1–0.9)	0.02
	Never	7/192	3.7	17/180	9.4	0.5 (0.2–1.2)	0.1
	Alcohol						
	Ever	8/155	5.2	19/165	11.5	0.5 (0.2–1.1)	0.07
	Never	5/163	3.1	12/153	7.8	0.4 (0.1–1.0)	0.06
	Coffee						
	Ever	12/267	4.5	28/296	9.5	0.5 (0.2–0.9)	0.03
	Never	1/51	2.0	3/22	13.6	0.2 (0.1–1.5)	0.1
Powers et al., 2006 [24]	All individuals	26/352	7.4	61/484	12.6	0.62 (0.38–1.01)	-
	Gender						
	Male	16/217	7.4	43/298	14.4	0.52 (0.28–0.97)	-
	Female	10/135	7.4	18/286	6.3	0.80 (0.35–1.83)	-
	Smoking						
	Ever	16/155	10.3	36/285	12.6	0.80 (0.43–1.49)	-
	Never	10/197	5.1	25/199	12.6	0.37 (0.17–0.80)	-
Leibson et al., 2006 [22]	All individuals	18/197	9.1	24/197	12.2	0.7 (0.4–1.4)	-
	Age at PD onset						
	<70	12/89	13.5	10/89	11.2	1.2 (0.5–3.0)	-
	≥70	6/108	5.6	14/108	13.0	0.4 (0.2–1.1)	0.05<P<0.1
Kessler, 1972	All individuals	17/228	7.5	29/228	12.7	0.55 (0.29–1.04)	-
	Gender						
	Male	12/122	9.8	14/122	11.5	0.84 (0.37–1.90)	-
	Female	5/106	4.7	15/106	14.2	0.30 (0.10–0.86)	P<0.05

**Table 4:** Subgroup analysis of the association between diabetes mellitus (DM) and Parkinson's disease.

Category of variables	Variables of study characteristics	Number of studies	OR (95% CI)	I^2^	P-value heterogeneity
Gender	Male	5	0.71 (0.40–1.23)	74%	0.004
	Female	5	0.79 (0.41–1.49)	68%	0.01
Geographic location	Europe	7	0.94 (0.69–1.28)	77%	0.0002
	North America (U.S.)	4	0.61 (0.45–0.83)	0	0.85
	Asia	3	0.54 (0.23–1.27)	68%	0.04
Source of the control	Hospital setting	7	0.88 (0.62–1.25)	72%	0.002
	General population	7	0.63 (0.40–0.99)	75%	0.0004
DM duration	<10 years	2	0.90 (0.63–1.30)	0	0.34
	≥10 years	2	1.27 (0.79–2.05)	0	0.59
Antidiabetes drug	Insulin prescription	2	1.18 (0.74–1.89)	0	0.93
Smoking	Ever	2	0.67 (0.40–1.13)	0	0.33
	Never	2	0.37 (0.21–0.66)	0	0.97
DM assessment	Medical record	6	0.92 (0.70–1.21)	72%	0.003
	Questionnaire	7	0.57 (0.39–0.85)	54%	0.04

In sensitivity analyses, we excluded PD patients and control subjects diagnosed with dementia or cerebrovascular disease prior to the index date. No significant difference was observed in the association with diabetes and PD incidence (OR 0.73; 95% CI 0.48–1.12) [8], . We investigated the impact of DM assessment on the estimate of odds risk as well. Except one study with no mention of DM assessment method [21], the negative correlation was stronger in studies that identified DM through self-report or questionnaire (OR 0.57; 95% CI 0.39–0.85) [8], [9], [11], [24], [25], [28], [29] than physician diagnosis or relevant criterion in medical record (OR 0.92; 95% CI 0.70–1.21)[10], [12], [22], [23], [26], [27] (Table 3). When we removed one study at a time and analyzed the rest of studies, the ORs altered only minimally. The ORs ranged from 0.73 (95% CI 0.52–1.01) after excluding the study by Becker et al. whose study carried the most weight [10] to 0.73 (95% CI 0.56–0.95) after excluding the study by Ho et al. whose study carried the least weight) [28]. In general, stratification of the studies by quality-associated variables did not obviously reduce the heterogeneity of effect estimates.

### Assessment of publication bias

**Figure 3:**
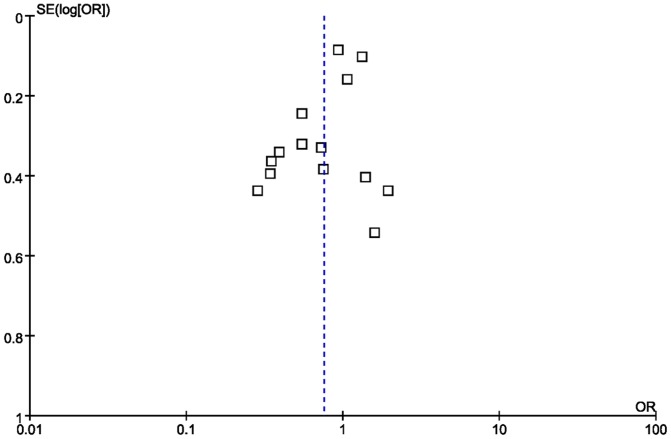
Funnel plot of diabetes and risk of Parkinson's disease.

The visual inspection of funnel plot indicated existence of publication bias to some extent (Figure 3).

## Discussion

### Summary of findings

Our updated systematic review suggested that diabetes may exert a lower risk of PD. In subgroup analyses, the negative correlation was still observed in studies from North America, non-PD control groups from general population, never smoking individuals, and DM ascertainment based on questionnaire or self-report. Stratification of gender and DM duration showed no significant association. No association was also found in European and Asian individuals, hospital-based controls, ever smoking subjects, DM assessment by medical record or physician diagnosis, and insulin prescription for DM, whereas oral anti-diabetes drug appeared to increase PD risk.

### Advantages

The issue of confounding is particularly a concern in a meta-analysis of observational studies when effect sizes are relatively small, as was the case in the studies considered [30]. Compared with the prior meta-analysis of 5 case-control studies [13], we incorporated 14 studies and performed subgroup analyses to explore the degree to which potential confounders may have influenced the findings, according to stratification on gender, geographic location, source of the control group, smoking, anti-diabetes drug prescription and duration of DM. In addition, some prospective cohort studies indicated the prevalence of PD among T2DM patients was 0.3% to 2.4% [4]–[7], [31], which was consistent with the most recently multicenter analysis (0.9%) [32]. The meta-analysis of cohort studies conducted by Cereda et al. also suggested that diabetes was a significant risk factor for the development of PD [13]. Thus, we have to take into account that the different results varying from a positive, null, or even to negative association between diabetes and the development of PD may be due to differences in study design and methodology. From the view of etiologic epidemiology, the use of cohort studies is preferable because of evidently causal hypothesis verification. However, cohort study is largely confined to practice because PD is age-related chronic disease with difficult recruitment, in addition to the required large populations and a long follow-up. Moreover, lost to follow up is inevitable to result in withdraw bias. Case-control studies may offer some advantages in diagnostic perspective, at least in terms of idiopathic PD diagnosis and of being less expensive and time-consuming for a larger mass of data collection. However, selective bias and recall bias are rooted in case-control design, and only associations, not real causal relations can be deduced.

### Limitations

This review has several limitations which should be addressed. First, all included case-control studies in the meta-analysis were relied on diagnostic criteria of primary studies included to identify idiopathic PD, regardless of chronic health status. However, diabetes patients are often comorbid with one or more chronic conditions and is also an important risk factor for cardiovascular disease with vascular parkinsonism. We have conduct sensitivity analysis by excluding participants with vascular type parkinsonism, and the results showed that no significant difference was observed in the association with diabetes and PD incidence. Since internationally accepted diagnostic criteria are not yet available, the responsibility of microvascular complications could not be thoroughly excluded [33]. Second, caution should be warranted in the overall estimates provided in this study as there was significant heterogeneity. Heterogeneity exists in terms of gender, geographical region, source of the control groups, DM assessment method, and adjustment for confounding factors. Despite the use of appropriate meta-analytic techniques with random-effect models, it could not account for these differences. Methodological variations that we were not able to test may have been an important factor. Third, our findings are based on the results of observational studies. We cannot exclude the possibility of potential confounding by various variables that may be associated with the exposure. Fourth, survival bias as a result of high mortality among diabetic patients could contribute to the inverse relationship between diabetes and PD in case-control studies. Finally, the possibility of publication bias is inherent in any meta-analysis of published data, because of small studies with null results tending not to be published. Publication bias may have resulted in an overestimate of the relationship between DM and risk of PD.

### Confounders consideration

Our finding of a lower risk of PD associated with DM raises questions as to the possible broad range of confounders across studies that may require more consideration to clarify this relationship. Firstly, two out of 14 included studies [12], [23] took into account the duration of diabetes with the cutoff of 10 years in the analyses for an increase of PD risk, and both indicated that no associations were found in cases and controls among diabetics. Although prospective studies are less prone to recall and selection biases, the results of two cohort studies are inconsistent. Driver et al. [5] reported a higher PD risk (RR 1.34) among diabetics who had diabetes for less than 10 years of the disease; however this finding may undermine the potential ascertainment bias, reverse causality, or common mechanisms that underlie both diabetes and PD. Inversely, Xu et al. [7] found higher PD risk (RR 1.75) among diabetics who had diabetes for more than 10 years at the time of baseline survey. In the present study, no statistical significance was found in subgroup analyses by DM duration [12], [23]. Future studies are warranted further to unveil the real association between the duration of diabetes and PD risk. Secondly, coffee and caffeine have been linked to both diabetes and PD. Meta-analysis studies have reported an inverse association between caffeine/coffee consumption and the risk of developing diabetes or PD [34], [35]. Thus, most primary studies used coffee consumption as adjusted factor in analyses [11], [23], [29]. However, D'Amelio et al. [11] reported that diabetes was consistently lower among PD cases compared with controls both in the coffee consumption in ever (at least a cup of coffee per week during their adult life) (OR 0.5; 95% CI 0.2–0.9) and in the coffee consumption in never (less than a cup of coffee per week during their adult life) (OR 0.2; 95% CI 0.1–1.5). Thirdly, while smoking is inversely associated with the risk of PD [36], active smoking is a known risk factor for diabetes [37]. When the relation between DM and PD was examined separately by smoking status, the association between DM and PD was stronger in non-smokers than in smokers, particularly a decrease of PD risk among diabetics in non-smoking men (OR 0.09; 95% CI 0.02–0.44) [24]. PD was in fact inversely associated with diabetes both among nonsmokers (less than an average of a pack of cigarettes per month during their adult life) (OR 0.5; 95% CI 0.2–1.2) and smokers (at least an average of a pack of cigarettes per month during their adult life) (OR 0.3; 95% CI 0.1–0.9) [11]. An effect of smoking was also not observed by Becker et al. [10] after stratification to category of smoking status (Current smoker: adjusted OR 0.94; 95% CI 0.39–2.27). In the present study, never smoking firmly strengthened the inverse association vs. no significant difference in ever smoking individuals in subgroup analyses. Therefore, an association between diabetes and PD did not affect by smoking status. In addition, smoking is an increment risk of type 2 diabetes, independent from possible confounders [38]. Smoking cessation programs are of great importance for primary care specialists dealing with diabetes. Unfortunately, smoking cessation can cause weight gain and a short-term worsening of some diabetic symptoms that may deter smokers with diabetes from attempting to quit [39]. Smoking status could be broken down by past, chronic and current. Thus, smoking cessation is a highly relevant subject for further research. Fourthly, while a few studies have shown an association between diabetes and a risk of certain cancer [40], there is a lower cancer risk among people with Parkinson's disease [41]. In the present study, 3 out of 14 studies mentioned an association between PD and certain cancers. Kessler [25] found that there were no differences in the reported prevalence of cancer or “tumors” among PD patients. Powers et al. [24] reported that the occurrence of any cancer was related to a slightly elevated PD risk in women (OR 1.34; 95% CI 0.75–2.39), but not in men (OR 0.99; 95% CI 0.63–1.53). Breast cancer in women showed no association with PD, and prostate cancer risk was associated with a slightly reduced PD risk (OR 0.77; 95% CI 0.33–1.79). No consistent association with other cancers were observed. Leibson et al. [22] found that PD with age at onset under 70 years was associated with increased likelihood of being assigned a diagnosis of all type of cancer; however, because of limitations in statistical power, the 95% confidence intervals were wide. However, no study focused on an association between cancer and PD among diabetics. Fifthly, two included studies showed that increased BMI had no significant association with PD onset [10], [23], while BMI was commonly recognized as a major anthropometric obesity indicator that have a substantial association with future diabetes risk [42], [43]. Conversely, D'Amelio et al. [11] reported that diabetes patients with a BMI of ≥ 26.1 kg/m^2^ have a negative association with PD incidence (OR 0.4; 95% CI 0.2–0.9), but no significant difference with a BMI of <26.1 kg/m^2^ (OR 0.4; 95% CI 0.1–1.3). Was PD in fact inversely associated with diabetes for a possible effect of BMI? Obesity paradox in diabetes mellitus was also found in mortality. Adults who are normal weight at the time of incident diabetes have higher mortality than adults who are overweight or obese [44]. Diabetes induced by the metabolic stress of obesity may be a fundamentally different problem from diabetes that develops in the absence of the stress of obesity among PD patients. Mechanisms to explain negative association between PD with comorbid diabetes and obesity are unknown.

### Public Health Implication

The incidence rate of PD among diabetes patients increased with age and was dramatically high in patients aged >65 years [1], [31], as age confirmed a proverbial effect modifier of increasing PD prevalence. Although age was not stratified by subgroups in the present review, the mean age of PD onset was over 60 years. Peroxisome proliferator-activated receptor γ coactivator 1α (PGC-1α, encoded by PPARGC1) was identified as a potential therapeutic target for early intervention in PD patients with diabetes[45], [46], and glucagon-like peptide-1 (GLP-1) and its analogues could also be possible treatments for cognitive deficits in individuals with neurodegenerative disorders [47], [48]. This meta-analysis suggested that insulin prescription of DM may not have much impact on the relationship between diabetes and risk of PD, whereas oral anti-diabetes drug appeared to increase PD risk.

### Biological Plausibility

The possible mechanisms underlying the association of diabetes with a decreased PD risk are the mutual pathophysiological interactions. First, the coexistence of dopaminergic neurons and insulin receptors in the substantia nigra suggested a direct association between the two diseases [49]. Second, sirtuins, an evolutionarily conserved class of seven proteins (SIRT 1–7) regulating a variety of cellular functions such as genome maintenance, longevity, and metabolism [50], possesses antagonizing effect on a target's activity despite having the same molecular target [51]. SIRT1 activators will exert their activity protecting individuals from diabetes [51], while inhibition of SIRT2 would protect dopaminergic cell against death both in *vitro* and in a drosophila model of PD [52]. Third, genetic susceptibility, lifestyle choices, and exposure to toxic environmental factors may lead to mitochondrial dysfunction [3], [53], endoplasmic reticulum (ER) stress [54], [55], inflammation [56]–[58], impaired glucose tolerance/insulin resistance[3], [52], [59], and metabolic dysregulation [60]. The dysregulation of these pathways may ultimately lead to neurodegenerative disease and/or diabetes [2], [3]. In the subgroup analysis according to geography location, we found that an inverse association between diabetes and the prevalence of PD in U.S. population, but discordant results obtained in Asia and Europe. This heterogeneity of results across populations with different ancestry boosts the possibility of a genetic influence. However, the biological mechanism behind the association of and PD risk and diabetic patients is far not clear.

## Conclusions

Diabetic individuals had a decreased incidence of PD despite heterogeneity in study design, geographic area, assessment of exposure and outcome, and control of potential confounders. More researches, both epidemiological and mechanistic, are warranted to clarify an understanding of the association between diabetes and risk of PD.

## Supporting Information

Appendix S1 **Search strategies.**
(DOC)

Appendix S2 **PRISMA checklist.**
(DOC)
