# Supplementary material for: Diabetes and Risk of Parkinson's Disease: An Updated Meta-Analysis of Case-Control Studies
Source: PLoS One. 2014 Jan 21;9(1):e85781. doi: 10.1371/journal.pone.0085781 (PMC3897520; doi:10.1371/journal.pone.0085781)
Supplement: Appendix S1 — Search strategies. (DOC) [file pone.0085781.s001.doc]

**Appendix S1. Search strategy for Pubmed**

1 exp diabetes mellitus/

2 diabet$.mp.

3 glucose intolerance/ or glucose tolerance / or insulin resistance

4 toleran$.mp.

5 or/ 1-4

6 exp PARKINSON DISEASE/

7 (Parkinsons AND disease).ti,ab

8 Parkinson*.ti,ab

9 or/ 6-8

10 case-control study.pt.

11 epidemiologic study/ or longitudinal study/ or retrospective study

12 study.ab.

13 groups.ab.

14 or/ 10-13

15 exp animals/ not humans.sh.

16 14 not 15

16 5 and 9 and 16
